# MCM2-7 complex is a novel druggable target for neuroendocrine prostate cancer

**DOI:** 10.1038/s41598-021-92552-x

**Published:** 2021-06-25

**Authors:** En-Chi Hsu, Michelle Shen, Merve Aslan, Shiqin Liu, Manoj Kumar, Fernando Garcia-Marques, Holly M. Nguyen, Rosalie Nolley, Sharon J. Pitteri, Eva Corey, James D. Brooks, Tanya Stoyanova

**Affiliations:** 1 Department of Radiology, Stanford University, 3155 Porter Drive, Palo Alto, CA 94304 USA; 2 Canary Center at Stanford for Cancer Early Detection, Stanford University, Palo Alto, CA USA; 3 Department of Urology, University of Washington, Seattle, WA USA; 4 Department of Urology, Stanford University, Stanford, CA USA

**Keywords:** Prostate cancer, Targeted therapies

## Abstract

Neuroendocrine prostate cancer (NEPC) is a lethal subtype of prostate cancer that rarely develops de novo in primary tumors and is commonly acquired during the development of treatment resistance. NEPC is characterized by gain of neuroendocrine markers and loss of androgen receptor (AR), making it resistant to current therapeutic strategies targeting the AR signaling axis. Here, we report that MCM2, MCM3, MCM4, and MCM6 (MCM2/3/4/6) are elevated in human NEPC and high levels of MCM2/3/4/6 are associated with liver metastasis and poor survival in prostate cancer patients. MCM2/3/4/6 are four out of six proteins that form a core DNA helicase (MCM2-7) responsible for unwinding DNA forks during DNA replication. Inhibition of MCM2-7 by treatment with ciprofloxacin inhibits NEPC cell proliferation and migration in vitro, significantly delays NEPC tumor xenograft growth, and partially reverses the neuroendocrine phenotype in vivo. Our study reveals the clinical relevance of MCM2/3/4/6 proteins in NEPC and suggests that inhibition of MCM2-7 may represent a new therapeutic strategy for NEPC.

## Introduction

Androgen deprivation therapy has been the standard therapy for advanced and metastatic prostate cancer since the middle of the last century. While androgen deprivation therapy is temporarily effective, almost invariably the cancers evolve strategies to grow at low levels of androgens, a state known as castration resistant prostate cancer (CRPC)^1^. Neuroendocrine prostate cancer (NEPC) accounts for 10–20% of CRPC^2–5^. While rare, NEPC can also arise de novo as primary tumors^2–5^. NEPC is commonly characterized by expression of neuroendocrine markers, aggressive clinical behavior, and loss of expression of the androgen receptor (AR) leading to resistance to therapies that target the AR pathway^2–5^. Currently, there are no long-term effective treatments for NEPC, underlining the urgent unmet clinical need to identify new therapeutic strategies for NEPC.

The minichromosome maintenance (MCM) complex is a DNA helicase that plays an important role in DNA replication. MCM complex is assembled as a double-hexamer ring structure by six MCM proteins, MCM2 to MCM7 (MCM2-7)^6^. The MCM complex regulates DNA replication and genome stability by unwinding DNA double strands and facilitating replication fork progression. MCM proteins have been used as biomarkers for rapid cell proliferation in clinicopathological diagnosis and prognosis as well as tumor progression markers in multiple cancer types^7^. For example, high levels of MCM2 are associated with poor survival in patients with prostate, breast, and lung cancers^8–10^, while high MCM3 expression correlates with worse survival in patients with brain cancer^11^. MCM4 expression is also associated with aggressive behavior in breast and lung cancers^12,13^. Up-regulation of MCM6 is observed in metastatic CRPC and correlates with recurrence and metastasis of hepatocellular carcinoma^14,15^.

Here, we demonstrate that MCM2/3/4/6 are significantly elevated in cell line and patient-derived xenograft (PDX) models of NEPC and, most importantly, in patient NEPC samples across three independent metastatic prostate cancer clinical datasets. We further demonstrate that inhibition of MCM2-7 via ciprofloxacin suppresses NEPC cell proliferation, colony formation, and cancer cell invasion in vitro. Moreover, treatment with ciprofloxacin leads to a significant decrease in tumor growth of NEPC xenografts and decrease in the levels of NEPC markers in vivo. Our findings demonstrate that MCM2/3/4/6 levels are elevated in NEPC and inhibition of MCM2/3/4/6 potentially represents a promising therapeutic strategy for the lethal NEPC.

## Results

### MCM2/3/4/6 are upregulated in NEPC

**Figure 1:**
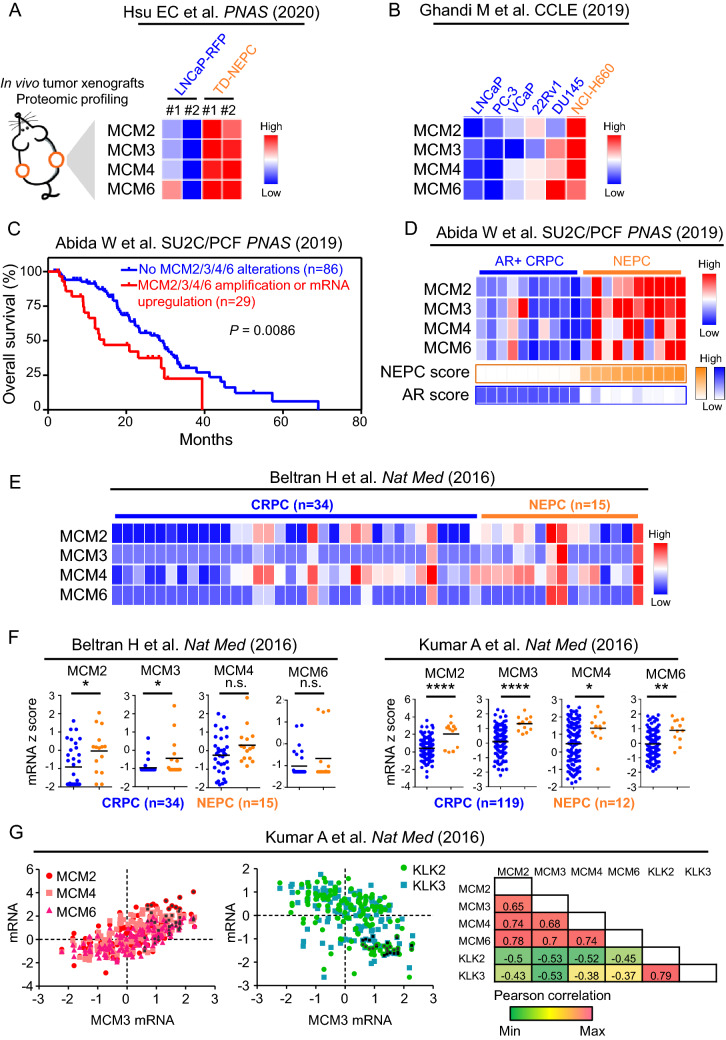
Identification of elevated levels of MCM2/3/4/6 in NEPC. (**A**) Heatmap depicting relative protein levels of MCM2/3/4/6 in Trop2-driven neuroendocrine prostate cancer (TD-NEPC) xenografts measured by label-free proteomics^16^. (**B**) mRNA levels of MCM2/3/4/6 were compared across multiple prostate cancer cell lines in CCLE database^17^. (**C**) Survival of patients with advanced prostate cancer from the SU2C/PCF dataset^18^ with no alterations in MCM2/3/4/6 (n = 86) compared to those with gene amplifications or mRNA upregulation (> twofold) in at least one of the MCM proteins (MCM2/3/4/6) (n = 29). (**D**) Heatmap depicting mRNA expression levels of MCM2/3/4/6 in the top 10 patient samples stratified by NEPC or AR scores extrapolated from the SU2C/PCF dataset^18^. (**E**) Heatmap depicting mRNA expression levels of MCM2/3/4/6 in NEPC (n = 15) versus CRPC (n = 34) patient samples from total of 10 NEPC patients and 25 CRPC patients from Beltran H et al.^19^. (**F**) To demonstrate quantitative differences, mRNA expression levels of MCM2/3/4/6 in NEPC (n = 15) versus CRPC (n = 34) patient samples and NEPC (n = 12) versus CRPC (n = 119) from Beltran H et al.^19^ (left) and Kumar et al. (right)^20^ are shown as dot plots. (**G**) Scatter plot of mRNA z-scores shows a correlation between mRNA levels of MCM3 and MCM2, MCM4, and MCM6 and inverse correlation of MCM3 with KLK2 and KLK3. Pearson correlations shown in the right table.

**Figure 2:**
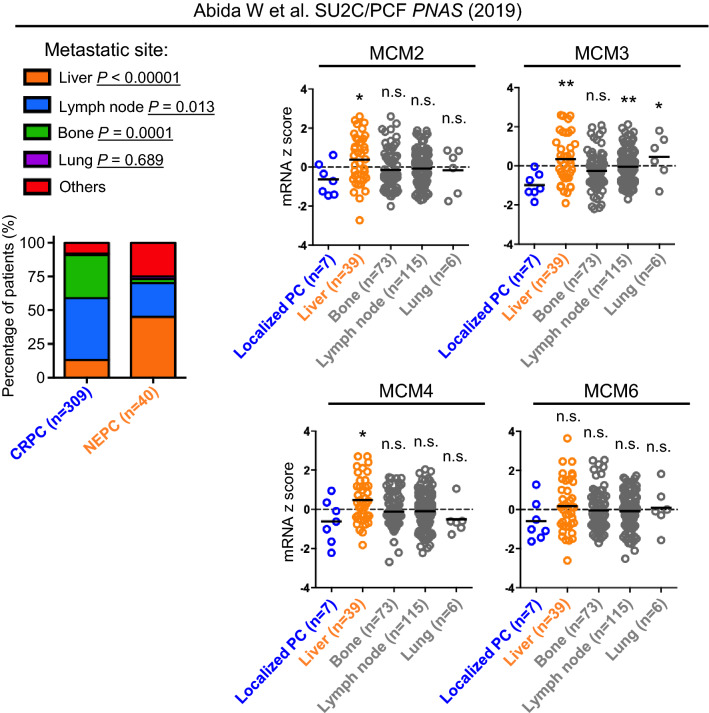
Elevated levels of MCM2/3/4 are associated with liver metastasis. (**A**) Metastatic incidence by sites from CRPC and NEPC from SU2C/PCF dataset^18^ was plotted as percentage of patients. The *p* values of the proportion differences of metastatic sites between CRPC and NEPC were calculated by z-score test for two population proportions. (**B**) Relative mRNA expression levels of MCM2/3/4/6 in different metastatic sites including liver, lymph node, bone, and lung from SU2C/PCF dataset^18^ are shown. *p < 0.05, **p < 0.01, and n.s. = not significant, determined by Student’s *t* test between localized prostate cancer with metastatic tumors.

To identify clinically relevant, druggable targets for NEPC, we analyzed our previously reported proteomic analysis of a NEPC model driven by the Trop2 oncogene (TD-NEPC)^16^ (Fig. 1A). Four proteins of the MCM2-7 complex, including MCM2/3/4/6, were identified as highly elevated in the TD-NEPC model when compared to LNCaP prostate adenocarcinoma xenografts (Fig. 1A). Elevated gene expression levels of MCM2/3/4/6 were also observed in NCI-H660, a NEPC cell line, when compared to prostate adenocarcinoma cell lines and two CRPC cell lines, 22Rv1 and DU145, showed slight up-regulation of MCM2/3/4/6 genes compared with LNCaP, VCaP, and PC-3 cell lines^17^ (Fig. 1B). Moreover, CRPC with either MCM2/3/4/6 gene amplifications or > twofold mRNA up-regulation were associated with worse patient overall survival^18^ (Fig. 1C and Supplementary Fig. 1). Gene expression levels of MCM2/3/4/6 were also specifically elevated in human NEPC when compared to adenocarcinoma CRPC^19,20^ (Fig. 1D-F). Likewise, a positive correlation of mRNA expression levels of MCM2, MCM4 and MCM6 with MCM3, and an inverse correlation with AR downstream targets KLK2 and KLK3 (PSA) was observed^20^ (Fig. 1G). Furthermore, relatively higher mRNA levels of MCM2/3/4 were associated with liver metastasis when compared with localized prostate cancer, which may be attributed to the higher incidences of liver metastasis in NEPC when compared to adenocarcinoma CRPC in the SU2C/PCF cohort^18^ (Fig. 2).

**Figure 3:**
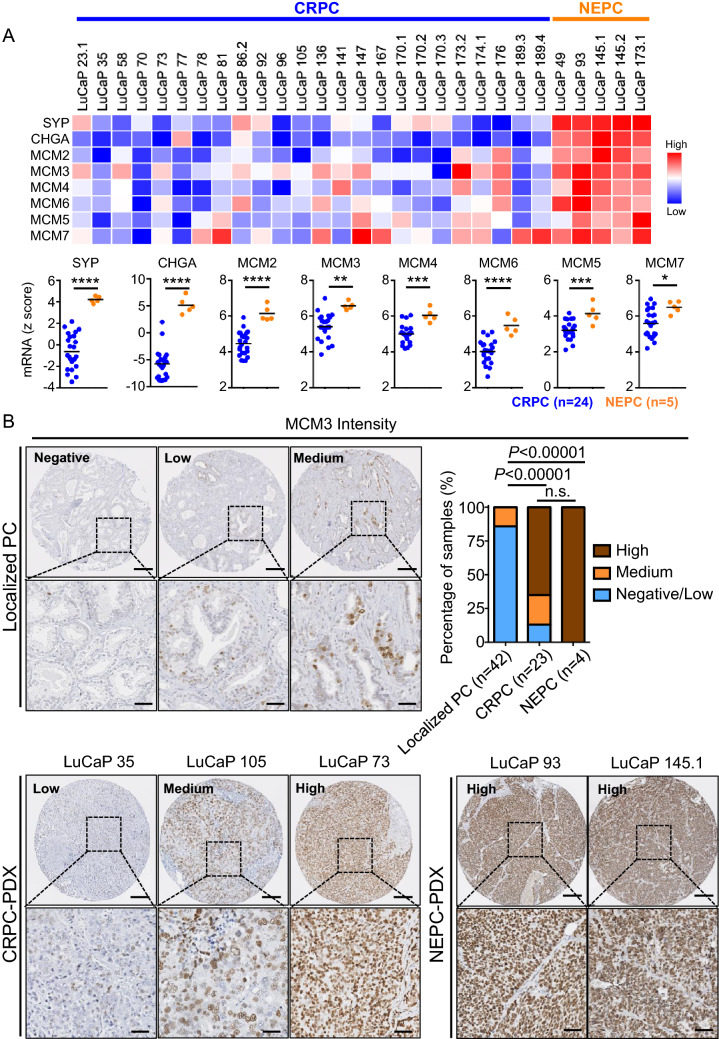
MCM2/3/4/6 are elevated in NEPC patient derived xenografts (PDX). (**A**) mRNA z scores of MCM2/3/4/5/6/7 and neuroendocrine markers (SYP and CHGA) in NEPC (n = 5) versus CRPC (n = 24) tissue samples from PDXs are shown as heatmap and scatter plots. *p < 0.05, **p < 0.01, ***p < 0.001 and ****p < 0.0001, determined by Student’s *t* test. (**B**) MCM3 IHC in localized prostate cancer and PDX TMAs is shown. Scale bars represent 200 µm and 50 µm respectively. The intensity of IHC staining for MCM3 was scored as negative, low, medium, and high and plotted. *p* < 0.00001 by z-score test for two population proportions.

**Figure 4:**
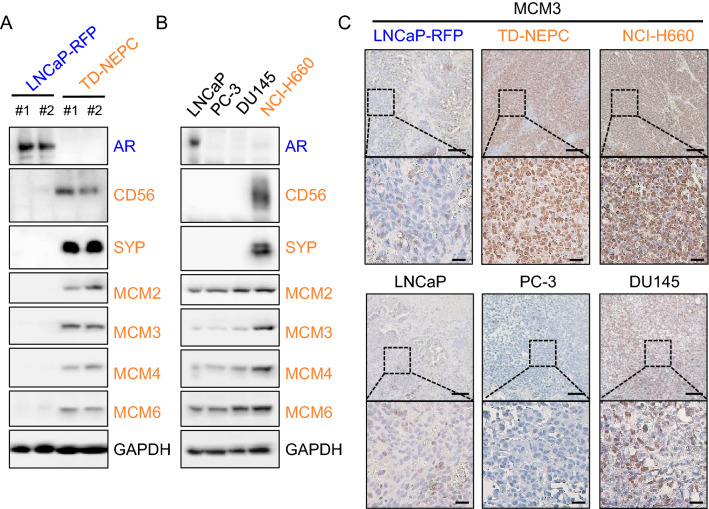
Protein levels of MCM2/3/4/6 are elevated in NEPC cell lines. (**A**) Protein levels of MCM2/3/4/6, AR, two neuroendocrine markers (CD56 and SYP), and GAPDH in LNCaP-RFP and TD-NEPC xenografts were measured by western blot. (**B**) Western blots of MCM2/3/4/6, AR, CD56, SYP, and GAPDH in LNCaP, PC3, DU145, and NCI-H660 (neuroendocrine) prostate cancer cell lines. (**C**) IHC staining for MCM3 in xenograft tumors derived from prostate cancer cell lines. Representative images are shown. Scale bars represent 100 and 20 microns respectively.

Consistent with our results in human NEPC, we found elevated mRNA levels of MCM2/3/4/6 as well as MCM5 and MCM7 in NEPC PDXs^21^, (Fig. 3A). We performed immunohistochemical (IHC) analysis of PDX tissue microarrays which further confirmed the expression of MCM3, MCM4, MCM5, MCM6, and MCM7 at the protein level in NEPC PDXs (Fig. 3B and Supplementary Figs. 2 and 3). In addition, MCM2/3/4/6 protein levels were also notably elevated in the NEPC cell line models TD-NEPC and NCI-H660 compared to prostate adenocarcinoma cell lines (Fig. 4A,B and Supplementary Figs. 4 A and B). IHC staining showed high levels of MCM3 in NEPC cell line tumor xenografts when compared to prostate adenocarcinoma cell line xenografts (Fig. 4C). Taken together, our results demonstrate that high levels of MCM2-7 complex is associated with NEPC.

### NEPC is sensitive to MCM inhibition

**Figure 5:**
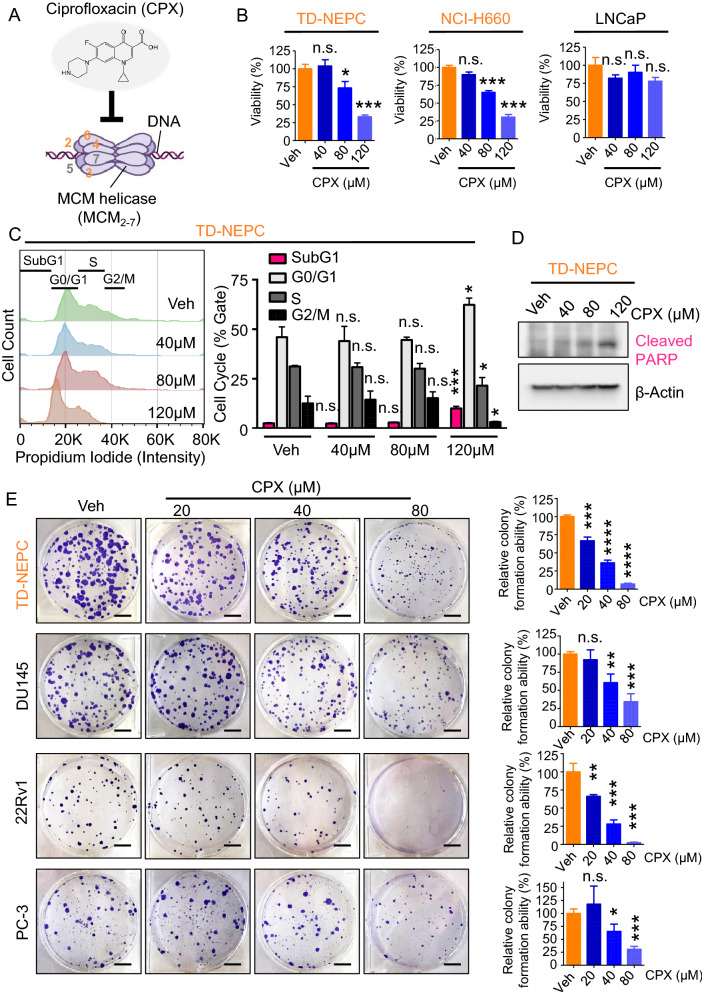
MCM inhibition via Ciprofloxacin inhibits NEPC growth in vitro. (**A**) Structure of ciprofloxacin (CPX) and cartoon illustration of the MCM2-7 double hexamer complex. The cartoon was drawn in BioRender (https://biorender.com/). (**B**) Viability of TD-NEPC, NCI-H660, and LNCaP cells with ciprofloxacin treatment. (**C**) Cell cycle analysis (SubG1, G0/G1, S, and G2/M phase as indicated) of TD-NEPC cells with ciprofloxacin treatment by propidium iodide staining. Relative cell counts per condition (%) were quantified and plotted (right graph). (**D**) Western blots of cleaved PARP (apoptosis marker) and β-actin in TD-NEPC cells with ciprofloxacin treatment for 72 h. (**E**) Colony formation assay of TD-NEPC, DU145, PC-3, and 22Rv1 cells with ciprofloxacin treatment. Scale bar represents 1 cm. Relative colony formation ability (%) was quantified, normalized to vehicle control, and plotted (right graph). All experiments were performed in triplicates and two independent biological replicates. Error bars represent standard deviation. *p < 0.05, ***p < 0.001, and n.s. = not significant, determined by Student’s *t* test. (**A**) Structure of ciprofloxacin (CPX) and cartoon illustration of the MCM2-7 double hexamer complex. (**B**) Cell viability assay of TD-NEPC and NCI-H660 NEPC cells treated with the indicated doses of ciprofloxacin. (**C**) Colony formation assay of TD-NEPC with ciprofloxacin treatment. Scale bar represents 1 cm. Relative colony formation ability (%) was quantified, normalized to vehicle control, and plotted (right graph). (**D**) Ciprofloxacin inhibits tumorsphere formation in 3D culture of TD-NEPC cells. Scale bar = 100 microns. Number of spheres per well is plotted (right graph). (**E**) Matrigel drop 3D invasion assay of TD-NEPC cells treated with ciprofloxacin (20 and 40 μM) or vehicle control. Scale bar = 200 microns. All experiments were performed in triplicate. Error bars represent standard deviation. *p < 0.05, ***p < 0.001, and n.s. = not significant, determined by Student’s *t* test.

The MCM2-7 complex is assembled as a double hetero-hexamer of MCM2 to MCM7 (Fig. 5A). The complex functions as a DNA helicase to unwind DNA replication forks at the beginning of DNA replication. Ciprofloxacin is an FDA approved antibiotic previously shown to inhibit MCM2-7 DNA helicase activity in vitro (Fig. 5A)^22^. We tested whether inhibition of MCM helicase activity by ciprofloxacin would affect NEPC cell growth. NEPC cells, including TD-NEPC and NCI-H660 cells, showed lower viability in a dose-dependent manner to ciprofloxacin while a prostate adenocarcinoma model (LNCaP) was unaffected (Fig. 5B). We further evaluated cell cycle and apoptosis by measuring DNA content (propidium iodide staining) and cleaved PARP1 by western blot. Ciprofloxacin slowed down the cell cycle at G0/G1 phase, increased the percentage of dead cells in the Sub G1 phase (Fig. 5C) and apoptosis marker, cleaved PARP1 (Fig. 5D and Supplementary Fig. 4C). Furthermore, treatment with ciprofloxacin significantly delayed the growth of TD-NEPC cells with a high level of MCMs and three CRPC cell lines with a mid-level of MCMs (DU145, PC-3, and 22Rv1) in a dose-dependent manner in colony formation (Fig. 5E). TD-NEPC and 22Rv1 cells were more sensitive to ciprofloxacin when compared to DU145 and PC3 depicted by their growth inhibition at 20 µM ciprofloxacin (Fig. 5E). Ciprofloxacin also inhibited 3D tumorsphere formation and invasion ability of TD-NEPC cells, while DU145 was less sensitive to ciprofloxacin in 3D tumorsphere assay (Supplementary Fig. 5). Collectively, inhibition of MCM2-7 DNA-helicase activity with ciprofloxacin dramatically delayed cell proliferation, clonogenicity, and invasion of NEPC cells. Consistent with the heterogenous MCMs pattern in CRPC in PDXs (Fig. 3B, Supplementary Fig. 2 and 3), three CRPC cell lines with mid-level MCMs (DU145, PC-3, and 22Rv1) also responded to ciprofloxacin in colony formation assay.

### MCM inhibition delays NEPC tumor growth in vivo and partially reverses the neuroendocrine phenotype

**Figure 6:**
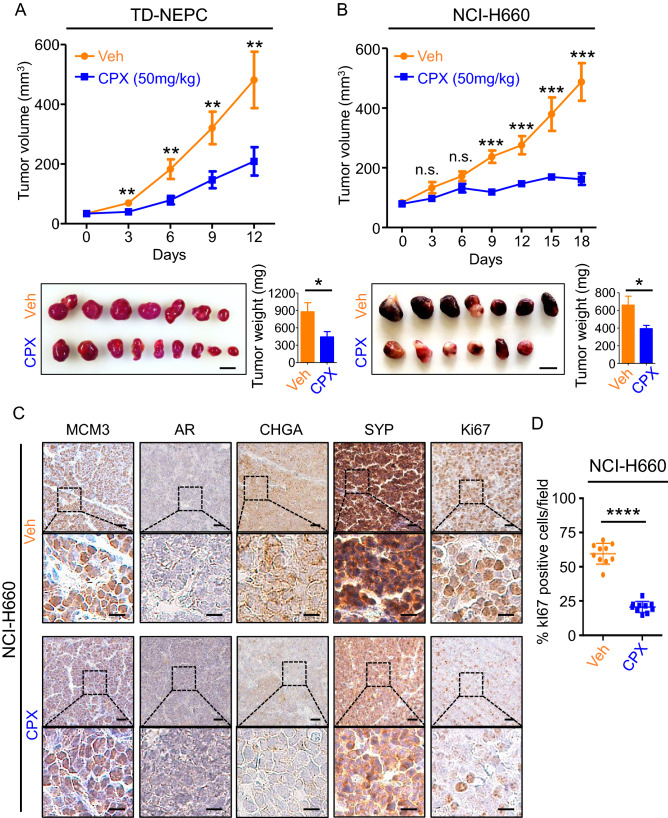
Ciprofloxacin inhibits NEPC tumor growth and suppresses expression of neuroendocrine markers in vivo. (**A**,**B**) Tumor growth curves, tumor images, and tumor weights of TD-NEPC (tumor n = 7–9) (**A**) and NCI-H660 (tumor n = 6–7) (**B**) treated daily with vehicle or ciprofloxacin (50 mg/kg via intraperitoneal injection). Error bars represent standard error of the mean (SEM). *p < 0.05, **p < 0.01, ***p < 0.001, and n.s. = not significant, determined by Student’s *t* test. (**C**) IHC staining for MCM3, AR, CHGA, SYP, and Ki67 in NCI-H660 tumor xenografts treated with vehicle (Veh) or ciprofloxacin (CPX). Scale bars = 25 microns (upper panels) and 10 microns (lower panels). (**D**) Percentage of Ki67-positive cells per image. Error bars represent standard deviation. ****p < 0.0001 by Student’s *t* test.

To test whether inhibition of MCM2-7 helicase activity would affect the growth of NEPC in vivo, we implanted subcutaneously two NEPC xenografts, TD-NEPC and NCI-H660, into immunocompromised male NSG mice. When the average tumor volumes reached 50–75 mm^3^, ciprofloxacin was administered intraperitoneally daily, and the tumor volumes and body weights were measured every 3 days. Ciprofloxacin significantly delayed tumor growth in both TD-NEPC and NCI-H660 NEPC models (Fig. 6A,B). Ciprofloxacin inhibits MCM2-7 activity, but not protein levels and as expected, MCM3 protein levels did not appear to change based on IHC staining. However, neuroendocrine markers (CHGA and SYP) and the proliferation marker (Ki67) showed significantly decreased staining after treatment with ciprofloxacin, while AR expression was not restored (Fig. 6C,D and Supplementary Fig. 6A). At the doses of ciprofloxacin (50 mg/kg) used in this study, toxicity assessed by loss of animal body above 80% was observed at Day 18 of treatment (Supplementary Fig. 6B and 2C). These results indicate that blocking the DNA helicase activity of the MCM2-7 complex using ciprofloxacin significantly delays NEPC tumor growth in vivo and partially reverses the expression of neuroendocrine markers.

## Discussion

Currently, there are no long-term effective therapeutic strategies for patients with NEPC. To gain insights into new actionable targets for NEPC, research efforts have been directed to identify key regulators of NEPC development and progression. MYCN and AURKA amplification, RB loss and TP53 mutations, upregulation of BCL2, as well as aberrant expression of transcription factors BRN2, FOXA2, and ONECUT2 have been associated with or implicated in NEPC development^23–33^. Here, we utilized proteomic analysis of a previously characterized NEPC model and identified MCM2, MCM3, MCM4, and MCM6 proteins as novel targets to inhibit NEPC growth. High levels of MCM2/3/4/6 were significantly enriched in human NEPC compared to castration-resistant prostate adenocarcinoma. NEPC can arise from neuroendocrine transdifferentiation of prostate adenocarcinoma and is orchestrated by global epigenetic modifications mediated by SOX2 and EZH2^27,34^. Genistein and trichostatin A (TSA), compounds that have been shown to act through altering epigenetic silencing suppress the expression of all MCM genes in prostate cancer^35–39^. These findings suggest that elevated levels of MCM2/3/4/6 in NEPC may be driven in part by upregulated SOX2 and EZH2 during NEPC development. The precise mechanism underlying the elevated levels of MCM2/3/4/6 and the functional role of MCM2/3/4/6 in NEPC development is yet to be elucidated.

Ciprofloxacin is an FDA approved antibiotic that is ubiquitously used to treat various bacterial infections and has been shown to inhibit bacterial DNA replication through inhibition of DNA gyrase and DNA topoisomerase. In addition, several studies have demonstrated the ability of ciprofloxacin to induce apoptosis, arrest cell cycle, and inhibit proliferation of human colon, lung, and prostate cancer cells^40–42^. In this study, we utilized ciprofloxacin to inhibit MCM2-7 activity in NEPC. We demonstrated that ciprofloxacin significantly delays NEPC cell growth and migration. Furthermore, inhibition of MCM2-7 activity via ciprofloxacin exhibits potent anti-tumor effects in NEPC, reverses neuroendocrine features and reveals a potential new clinically relevant target for NEPC.

Our findings uncover that MCM2/3/4/6 are markedly elevated in patient NEPC and represent new druggable targets for therapeutic intervention. The study further reveals that inhibition of MCM2-7 complex using ciprofloxacin or other targeted approaches may represent a new effective therapy for NEPC. Our studies warrant further exploration of design and screening for MCM2-7 complex-specific inhibitors.

## Methods

All methods were carried out in accordance with the relevant guidelines and regulations of Stanford University.

### Datasets

All the raw data were obtained from public datasets accessed through cBioPortal for Cancer Genomics (https://www.cbioportal.org/)^43^. Transcript levels of MCM2/3/4/6 in multiple prostate cancer cell lines were obtained from the Cancer Cell Line Encyclopedia (CCLE) dataset^17^. Putative copy number alterations, NEPC score, AR score, tumor site, mRNA levels of MCM2/3/4/6 in metastatic, castration-resistant prostate cancer, as well as patient overall survival data were collected from SU2C/PCF dataset^18^. Transcript levels of MCM2/3/4/6 in metastatic, castration-resistant adenocarcinoma (CRPC-Adeno), and NEPC (CRPC-NE) were acquired from a neuroendocrine prostate cancer dataset^19^. Transcript levels of MCM2/3/4/6 and KLK2/KLK3 in metastatic prostate cancer were obtained from Kumar et al.^20^. Transcript levels of MCM2/3/4/5/6/7 and SYP/CHGA (neuroendocrine markers) from PDXs were extrapolated from Nguyen et al.^21^.

### Heatmap, survival and correlation analyses

Heatmaps of mRNA z-scores were generated using Morpheus (https://software.broadinstitute.org/morpheus/). Kaplan–Meier survival analysis was performed using Prism 6.0 by comparing samples with no alterations of MCM2/3/4/6 (n = 86) to those with gene amplifications or mRNA upregulation (> twofold) in at least one of the MCMs (MCM2, MCM3, MCM4 or MCM6) (n = 29). Pearson correlation coefficient score of MCM2/3/4/6 and KLK2/KLK3 was acquired from the Fred Hutchinson CRC dataset accessed via cBioPortal Cancer Genomics^43^. A correlation heatmap table was used to visualize the individual association of each gene.

### Cell lines and cell culture conditions

Human prostate cancer cell lines, LNCaP, PC-3, DU145, 22Rv1, and NCI-H660, were purchased from the American Type Culture Collection (ATCC). A Trop2-driven neuroendocrine prostate cancer (TD-NEPC) cell line was generated as described previously by infection of LNCaP cells with a lentiviral vector expressing Trop2 and red fluorescent protein (RFP)^16^. LNCaP, PC-3, DU145, 22Rv1, LNCaP-RFP, and TD-NEPC cells were maintained in RPMI containing 10% FBS, 100 U/ml penicillin, and 100 μg/ml streptomycin. NCI-H660 cells were maintained in RPMI 1640 medium with 5% fetal bovine serum, 0.005 mg/ml insulin, 0.01 mg/ml transferrin, 30 nM sodium selenite, 10 nM hydrocortisone, 10 nM beta-estradiol, and 4 mM l-glutamine. Cell lines were authenticated by the Stanford Functional Genomics Facility (SFGF) based on Short Tandem Repeat (STR) profiling and were routinely checked for mycoplasma contamination by MycoAlert™ Mycoplasma Detection Kit (Lonza). Ciprofloxacin was purchased from Sigma (17850-5G-F).

### Western blotting

Cancer cells were collected and lysed in RIPA lysis buffer supplemented with protease and phosphatase inhibitors. Protein concentration was measured by BCA assay. Samples in SDS sample buffer were heat-denatured at 95 °C for 5 min. The proteins were separated by SDS-PAGE, transferred onto a nitrocellulose membrane, and blotted with primary antibodies including anti-MCM2 (sc-373702, 1:1000 dilution), anti-MCM3 (sc-390480, 1:1000 dilution), anti-MCM4 (sc-28317, 1:1000 dilution), anti-MCM6 (sc-393618, 1:1000 dilution), anti-AR (sc-7305, 1:1000 dilution), anti-SYP (sc-17750, 1:2000 dilution), anti-CD56 (sc-7326, 1:500 dilution), anti-cleaved PARP1 (sc-56196, 1:1000 dilution), anti-β-Actin (sc-47778, 1:2000 dilution), and anti-GAPDH (sc-47724, 1:2000 dilution) purchased from Santa Cruz Biotechnology. Secondary HRP conjugated antibody (PI31432, 1:6000 dilution) was purchased from Fisher Scientific. Western blot development and detection was performed using Pierce™ ECL Western Blotting Substrate (Thermo Fisher Scientific) and IVIS Lumina optical imaging system (PerkinElmer). All the raw images of Western blot were summarized in Supplementary Fig. 4.

### Immunohistochemical staining

Tissue microarrays (TMAs) available at Stanford University comprised of normal prostate tissues and prostate cancers of a spectrum of grades taken from formalin-fixed paraffin-embedded radical prostatectomy specimens were used to assess expression of MCM proteins in normal prostate tissues and localized cancer. TMAs of LuCaP patient-derived xenograft models of advanced prostate cancers were constructed from subcutaneous tumors, three tumors per PDX models and three punches per tumor as previously described^21^. TMAs were sectioned at 4 microns and were deparaffinized, rehydrated, and heated to 95 °C for 30 min in 10 mM sodium citrate (pH 6.0) for antigen retrieval. A five-minute incubation in 3% hydrogen peroxide in 1xPBS was used to block endogenous peroxidase activity, and 2.5% horse serum in 1xPBS was applied for 1 h to reduce non-specific background. Sections were incubated overnight at 4 °C with indicated primary antibodies. Anti-MCM3 (sc-390480), anti-MCM4 (sc-28317), anti-MCM5 (sc-165994), anti-MCM6 (sc-393618), anti-MCM7 (sc-9966), anti-AR (sc-7305), anti-SYP (sc-17750), anti-CHGA (sc-393941), and anti-Ki67 (sc-23900) primary antibodies were purchased from Santa Cruz Biotechnology and used at 1:100 dilution. Secondary antibodies were purchased from Vector Labs (MP-7452) and used according to the manufacturer’s recommendations. After counterstaining with hematoxylin, the slides were dehydrated, mounted with cover slips, and imaged by Leica DMi8 microscope or Hamamatsu NanoZoomer.

### Viability assay

5000 (TD-NEPC, and LNCaP) or 10,000 (NCI-H660) cancer cells were seeded in 96-well plates and allowed to attach overnight. The following morning, ciprofloxacin was added at the indicated concentrations. After 3 days of treatment, the viable cells were quantified by CellTiter-Blue® Reagent (Promega) and percentage viability was computed by comparison to vehicle control.

### Cell cycle analysis by DNA content (propidium iodide staining)

2 × 10^5^ TD-NEPC cells were seeded in 12-well plates and allowed to attach overnight. The next day, ciprofloxacin was added at the indicated concentrations. After 3 days of treatment, all the cells, including attached and floating cells in culture media, were collected, washed with PBS, and fixed with 70% ethanol at − 20 °C overnight. After fixation, the cells were centrifuged at 1000 rpm for 5 min, washed with PBS, and then resuspended in 0.5 ml propidium iodide staining solution (propidium iodide (20 μg/ml), RNase A (20 μg/ml), and Triton X-100 (0.1%) in PBS) for 15 min at 4 °C in the dark. After that, the cells were washed with PBS and resuspended in 0.5 ml PBS for cell cycle analysis by flow cytometry (Guava EasyCyte HT Flow Cytometer System, Millipore). The overlay histogram plot of multiple conditions was generated by FlowJo software (FlowJo 10.7.2; https://www.flowjo.com/).

### Colony formation assay

500 cells per well were seeded in 6-well plates for indicated number of days dependent on cell lines (TD-NEPC and DU145 for 9 days, PC-3 for 12 days, and 22Rv1 for 15 days). Every 3 days, medium was replaced with fresh medium containing ciprofloxacin (vehicle, 20, 40, and 80 μM). After 9 days, the colonies were fixed and stained with crystal violet. Relative colony formation ability (%) was quantified by measuring colony area per well, and measurements were normalized based on the colony area of vehicle control.

### Tumorsphere assay

500 cells mixed with 50% Matrigel were seeded in 24-well plates for 15 days. Medium containing the indicated concentrations of ciprofloxacin was exchanged every 3 days. Tumorspheres were imaged at day 15 using a Leica stereomicroscope, and quantification was conducted using ImageJ (ImageJ 1.53e; https://imagej.nih.gov/ij/index.html) software by measuring the number of tumorspheres per well based on the RFP reporter signal.

### 3D Matrigel drop invasion assays

As previously described^16^, a 3D invasion assay was performed in 24-well plates using 5 × 10^4^ cancer cells in 10 μl of 100% Matrigel plated as a drop into each well. Imaging was performed on Day 0 and Day 6 using a Celigo Imaging Cytometer (Nexcelom Bioscience). The medium containing vehicle or the indicated concentrations of ciprofloxacin was changed every 3 days. Cell migration area outside of the drop was measured. The relative invasion ability of cancer cells was normalized to the vehicle controls. The orange pseudo-color represents living cells due to RFP fluorescent signals produced by the cell lines.

### Effects of ciprofloxacin on xenograft tumor growth in vivo

All animal experimental procedures were approved by the Institutional Animal Care and Use Committee (IACUC) of Stanford University and in accordance with ARRIVE guidelines. TD-NEPC (1 × 10^6^) or NCI-H660 (2 × 10^6^) cancer cells were mixed with 100 μl of Matrigel and implanted subcutaneously into both flanks of male NOD/SCID/IL-2Rγnull (NSG) mice. Before the average tumor volume reached 50–75 mm^3^, mice were randomized to receive either vehicle (0.1 N HCl/H_2_O) or ciprofloxacin (50 mg/kg dissolved in 0.1 N HCl/H_2_O) i.p. daily. Every three days, the tumor length (L), width (W), and height (H), as well as the mouse body weights were measured. Tumor volumes were calculated by the equation (LxWxH/2). Tumors were harvested, weighed, fixed with 10% buffered formalin, and embedded in paraffin for immunohistochemical staining.

## Supplementary Information

Supplementary Information.
