# Supplementary material for: MCM2-7 complex is a novel druggable target for neuroendocrine prostate cancer
Source: Sci Rep. 2021 Jun 25;11:13305. doi: 10.1038/s41598-021-92552-x (PMC8233352; doi:10.1038/s41598-021-92552-x)
Supplement: Supplementary file 1 — Supplementary Information. [file 41598_2021_92552_MOESM1_ESM.pdf]

SUPPLEMENTARY INFORMATION FOR:

MCM2-7 complex is a novel druggable target for neuroendocrine prostate cancer

Authors: En-Chi Hsu[1,2], Michelle Shen[1,2], Merve Aslan[1,2], Shiqin Liu[1,2], Manoj Kumar[1,2], Fernando Garcia-Marques[1,2], Rosalie Nolley[2,4], Holly M. Nguyen[3], Sharon J. Pitteri[1,2], Eva Corey[3], James D. Brooks[2,4], and Tanya Stoyanova[1,2*]

Affiliations: [1]Department of Radiology, Stanford University, Stanford, California, [2]Canary Center at Stanford for Cancer Early Detection, Stanford University, Palo Alto, California, [3]Department of Urology, University of Washington, Seattle, Washington, [4]Department of Urology, Stanford University, Stanford, California.

Supplementary Figure 1

Comparison of Survival Curves	
Log-rank (Mantel-Cox) Test	
Chi square	6.896
df	1
P value	0.0086
P value summary	**
Are the survival curves sig different?	Yes
Gehan-Breslow-Wilcoxon Test	
Chi square	8.813
df	1
P value	0.0030
P value summary	**
Are the survival curves sig different?	Yes
Median survival	
Up-regulation feature (n=29)	14.29
Non-up-regulation feature (n=86)	28.48
Ratio	0.5017
95% CI of ratio	-0.08792 to 1.091
Hazard Ratio	
Ratio	2.402
95% CI of ratio	1.249 to 4.621

Supplementary Figure 1. Statistical analysis for Figure 1C. Log-rank (Mantel-Cox) test, Gehan-Breslow-Wilcoxon test, median survival, and hazard ratio, were performed to compare the no alterations in all MCM2/3/4/6 (n=86) and gene amplification or mRNA upregulation in at least one of the MCMs (MCM2/3/4/6) (n=29).

Supplementary Figure 2

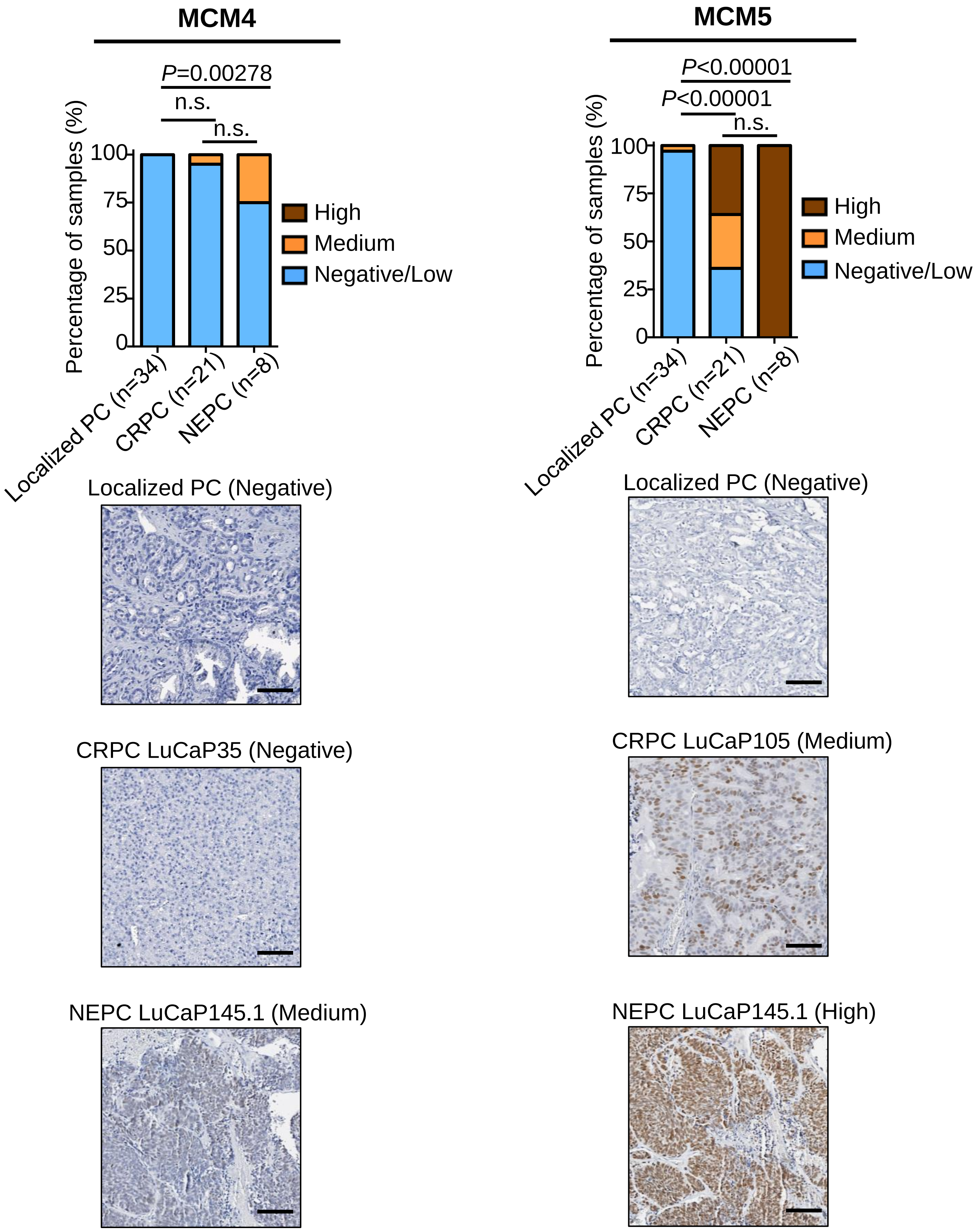

Supplementary Figure 2. Protein levels of MCM4 and MCM5 in patient samples and patient derived xenografts (PDX). Analysis of protein intensity of MCM4 and MCM5 in localized prostate cancer and CRPC/NEPC PDX TMAs is shown. P values comparing two groups were calculated (medium versus low for MCM4 staining; high versus medium and low for MCM5 staining) by z-score test for two population proportions. Intensity of IHC staining was scored as negative, low, medium, and high as shown. Scale bars represent 100 µm.

Supplementary Figure 3

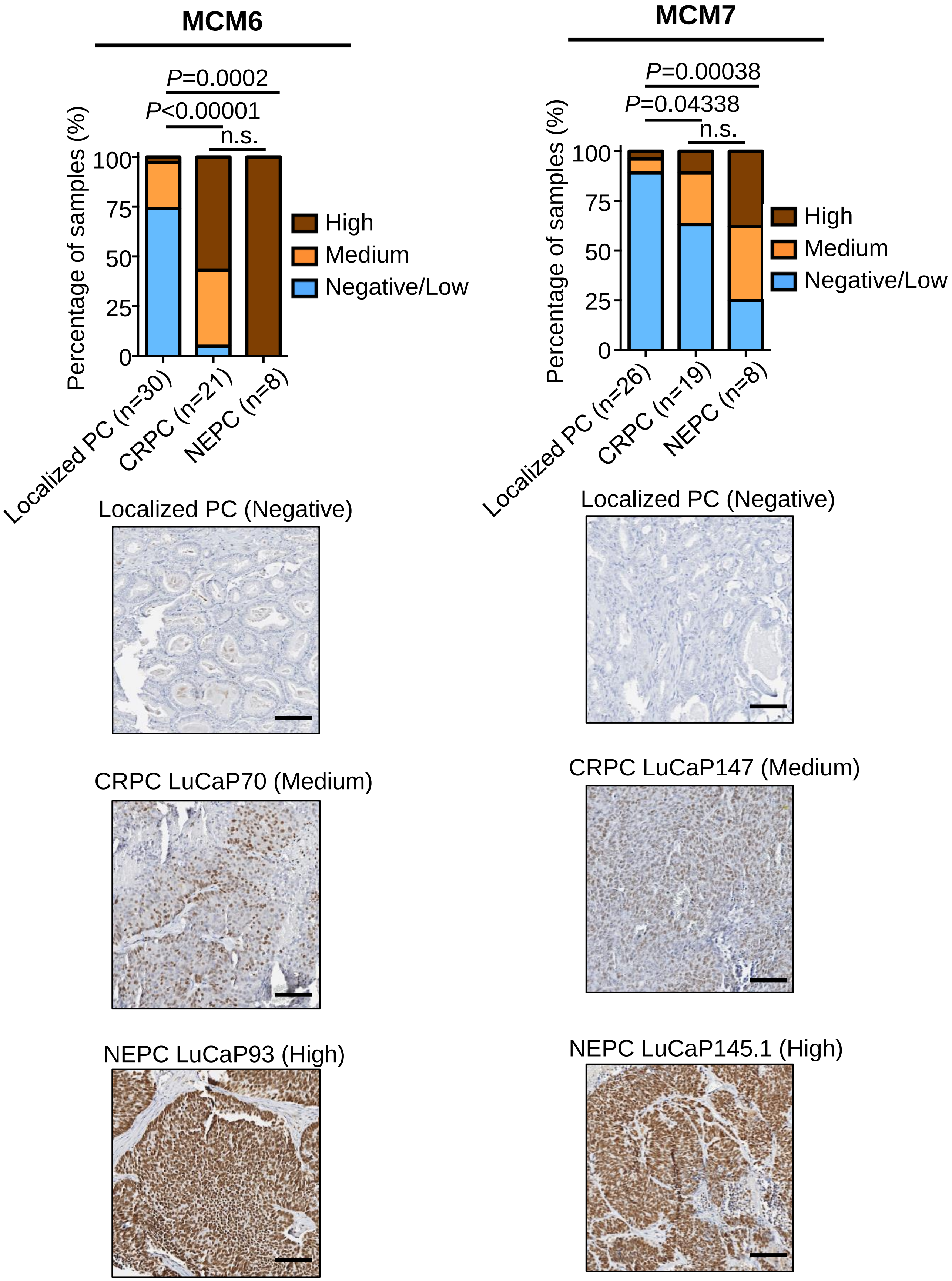

Supplementary Figure 3. Protein levels of MCM6 and MCM7 in patient samples and patient derived xenografts (PDX). Analysis of protein levels of MCM6 and MCM7 by IHC in localized prostate cancer and CRPC/NEPC PDX TMAs. P values comparing two groups were calculated and shown (high versus medium and low) by z-score test for two population proportions. Intensity of IHC staining was scored as negative, low, medium, and high as depicted in the represented images. Scale bars signify 100 µm.

Supplementary Figure 4

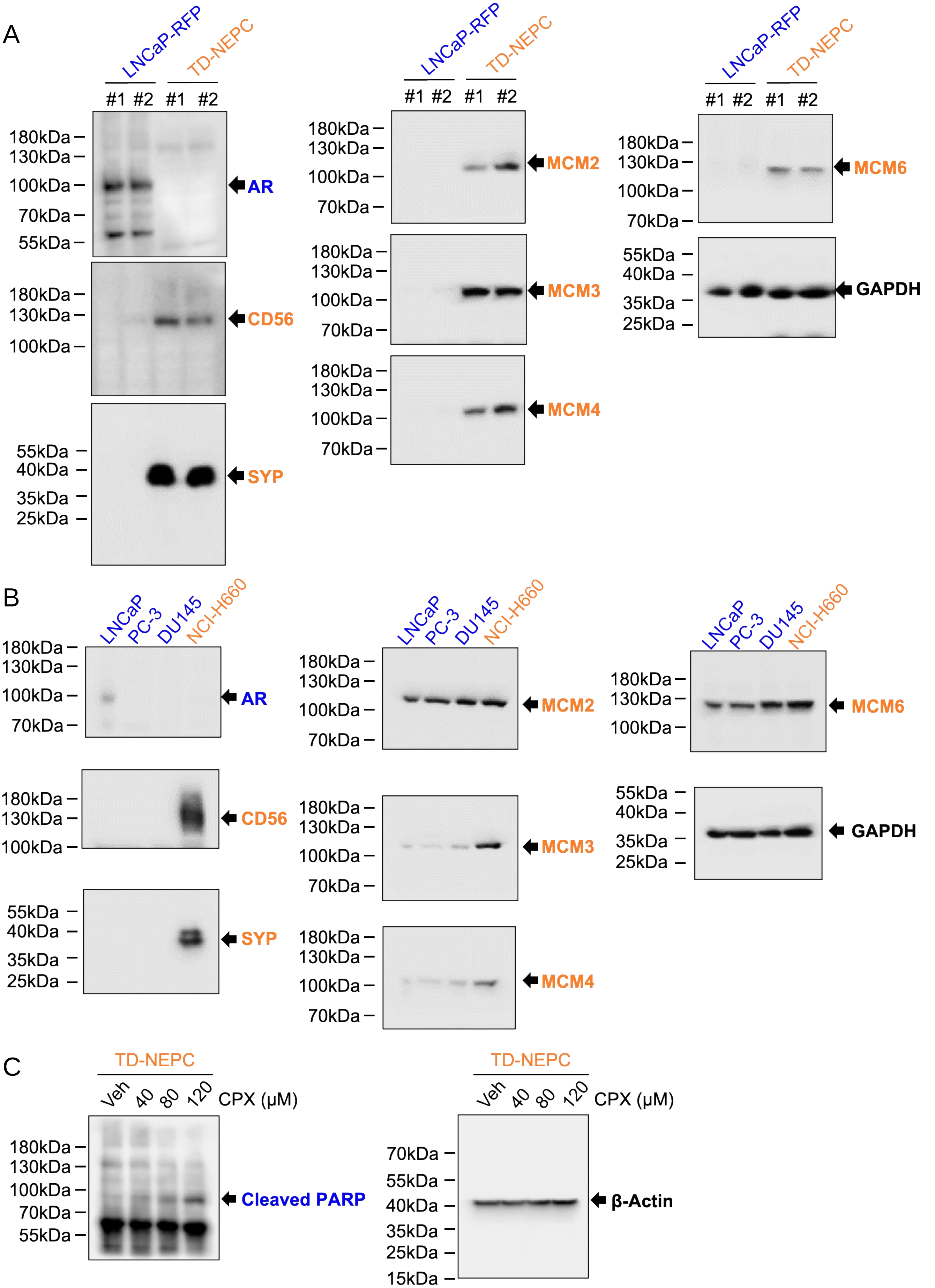

Supplementary Figure 4. Full western blot images. (A) Images used in Figure 4A. **(B)** Images used in Figure 4B. **(C)** Images were used in Figure 5D.

Supplementary Figure 5

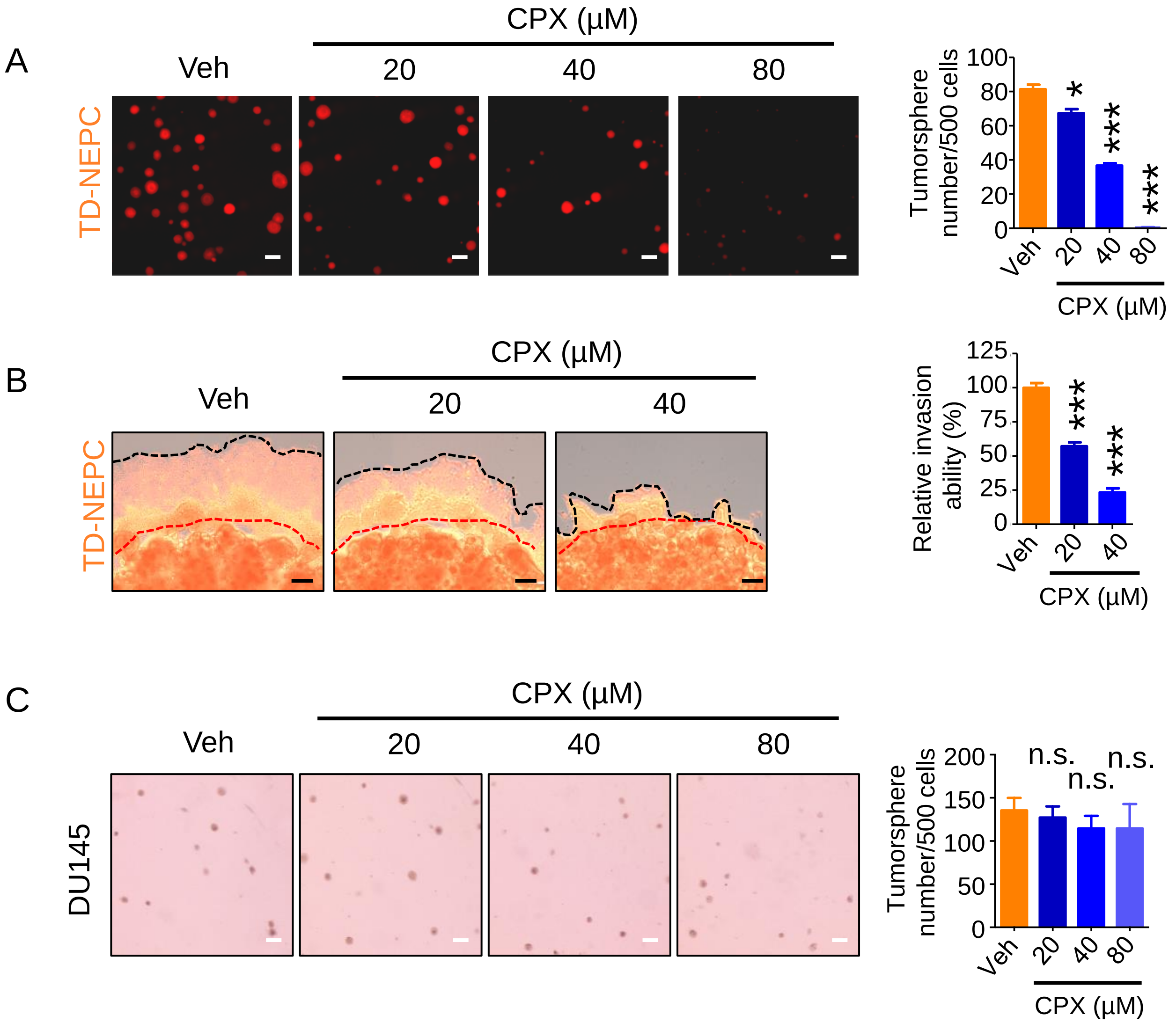

Supplementary Figure 5. Ciprofloxacin inhibits NEPC tumorsphere growth and invasion ability *in vitro*. (A) Ciprofloxacin inhibits tumorsphere formation in 3D culture of TD-NEPC cells. Scale bar = 100 microns. Number of spheres per well is plotted (right graph). **(B)** Matrigel drop 3D invasion assay of TD-NEPC cells treated with ciprofloxacin (20 and 40 µM) or vehicle control. Scale bar = 200 microns. **(C)** Ciprofloxacin dose not alter tumorsphere formation in 3D culture of DU145 cells. Scale bar = 100 microns. Number of spheres per well is plotted (right graph). All experiments were performed in technical triplicate and two independent biological replicates. Error bars represent standard deviation. *p < 0.05, ***p < 0.001, and n.s. = not significant, determined by Student's t-test.

Supplementary Figure 6

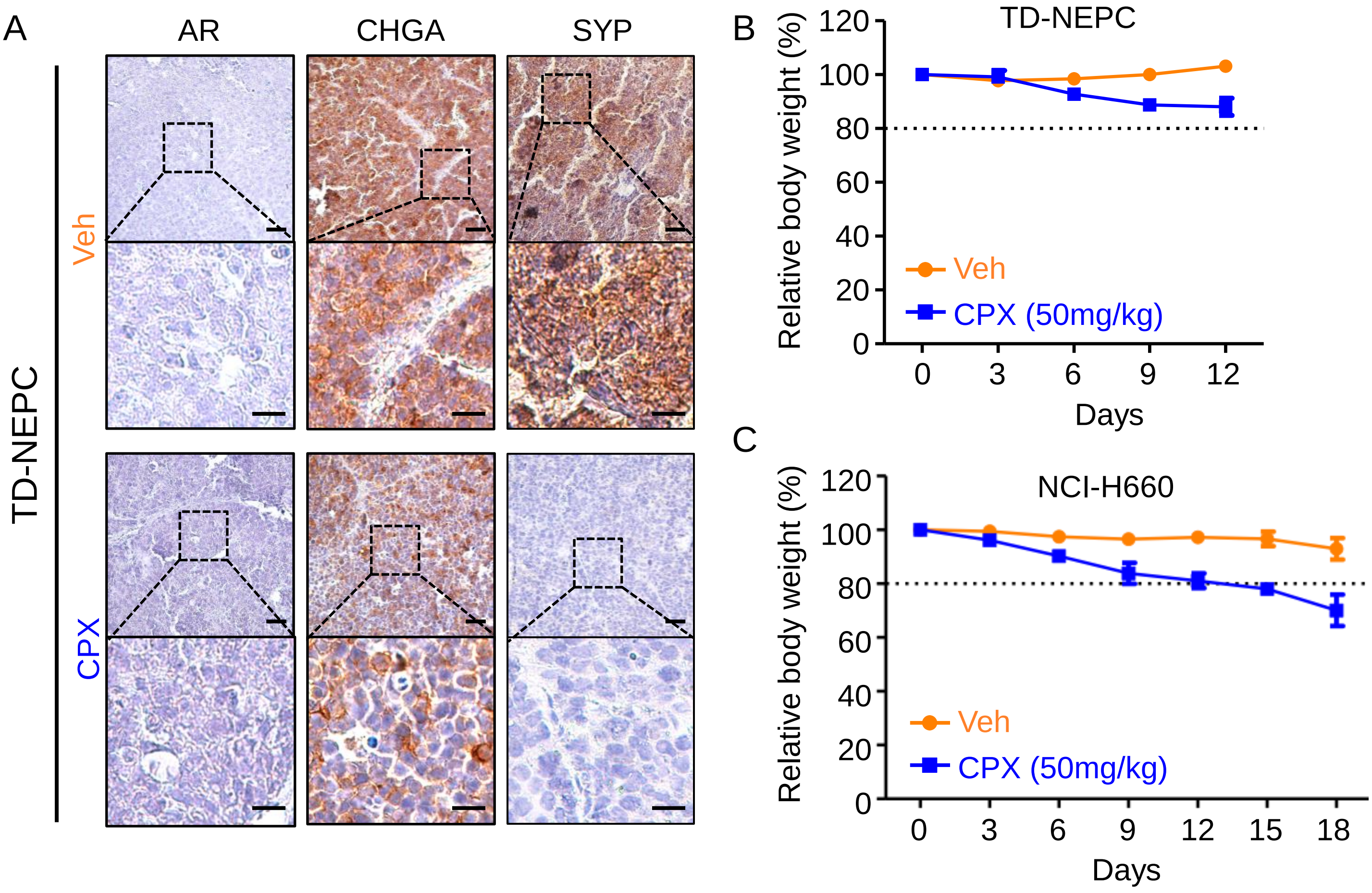

Supplementary Figure 6. Ciprofloxacin suppresses expression of neuroendocrine markers and has minimal measurable toxicity *in vivo*. (A) IHC staining for AR, CHGA, and SYP in TD-NEPC tumor xenografts from Figure 6A. Scale bar represents 25 microns (upper panel) and 10 microns (lower panel). **(B and C)** Plot of body weight of xenografted mice implanted with (Figure 6A) TD-NEPC (tumor n = 7-9) or (Figure 6B) NCI-H660 (tumor n = 6-7) shown in Figure 6.
